# Astragaloside IV alleviates GDM via regulating gut microbiota and gut microbiota metabolomic

**DOI:** 10.3389/fphar.2024.1431240

**Published:** 2025-01-06

**Authors:** Fengge Wang, Yongning Zhu, Hua Shu, Xiaoyun Zhang, Liting Duan, Dongmei Man, Yanping Wang

**Affiliations:** Department of Obstetrics, Affiliated Hospital of Jining Medical University, Jining Medical University, Jining, Shandong, China

**Keywords:** gut microbiota, gestational diabetes mellitus, astragaloside IV, metabolism, gut microbiota-related metabolites

## Abstract

**Background:**

Gestational diabetes mellitus (GDM), a severe pregnancy disorder, is a temporary form of diabetes that occurs during gestation. Astragaloside IV (AS IV), a natural and effective composition of *Astragalus membranaceus*, shows pharmacological effects against diabetes. On the contrary, the effects of AS IV on GDM development are still not clear. This study aims to investigate the role of AS IV in alleviating GDM in rats and determine whether AS IV exerts its anti-GDM properties through the regulation of gut microbiota and metabolite modulation.

**Methods:**

There were six pregnant SD rats in each of the four groups. First, the GDM model was induced by the streptozotocin (STZ, 45 mg/kg) injection on gestational days (GDs) 1–4, and AS IV intervention (10 mg/kg/d) was administered from 6 days before pregnancy until delivery. The measurements of relevant indicators pertaining to GDM symptoms and reproductive outcomes, along with the 16S rRNA sequencing data and LC-MS-based metabolomic profiles, were assessed across all groups.

**Results:**

After the 25-day intervention, the GDM model + AS IV group showed significantly decreased fasting blood glucose levels (*p* = 0.0003), mean insulin levels (*p* = 0.0001), and insulin resistance index (*p* = 0.0001). AS IV treatment also decreased the malformation rate (*p* = 0.0373) and increased the average fetal weight (*p* = 0.0020) of GDM rats. Compared to the control rats, GDM rats showed a significantly higher abundance of *Blautia* and *Anaerobiospirillum*. However, the dramatically elevated abundance of these microorganisms was markedly decreased by AS IV treatment. In contrast, compared to GDM rats without treatment, GDM rats treated with AS IV showed a significantly higher abundance of bacteria (*p* < 0.05), such as *Methanobrevibacter*, *Dubosiella*, and *Romboutsia*, which are beneficial to the rats. Additionally, we observed dramatically elevated production of metabolites, such as N-acetyl-l-leucine and lithocholic acid, after AS IV treatment through metabolomics analysis (*p* < 0.05). Furthermore, significant associations between most genera of gut bacteria and the altered levels of the metabolites connected to gut microbiota were also discovered.

**Conclusion:**

Our study demonstrated that AS IV could be an effective nutritional intervention strategy for targeting gut microbiota and metabolome profiles in GDM and provided experimental evidence supporting the use of AS IV to treat GDM.

## 1 Introduction

As one of the most serious and prevalent pregnancy complications, gestational diabetes mellitus (GDM) is induced by inadequate insulin production or signaling during pregnancy (Sweeting et al., 2022). GDM is a great threat to maternal health and fetal development, affecting approximately 5%–20% of all pregnant women worldwide (Gray et al., 2017). The high incidence of GDM and its possible adverse pregnancy outcomes are major challenges in obstetric practice. Therefore, safe and effective therapies for GDM are urgently needed.

Increasing evidence suggests that microbes residing in the human gut significantly participate in the regulation of health and processes of metabolism in the host (Moloney et al., 2014). Recently, it has been reported that an alteration of the gut microbiota is associated with diabetes (Ferrocino et al., 2018), GDM (Li et al., 2021), hypercholesterolemia (Mahajan et al., 2019), anxiety (Kelly et al., 2016), neurological diseases (Shin et al., 2020), and inflammatory bowel disease (Aldars-Garcia et al., 2021). Moreover, the microbiota residing in the gut obtains nutrients from the host, simultaneously promotes the digestion of complex nutrients, and regulates and balances metabolic functions (Jardon et al., 2022).

Gut dysbiosis reduces the relative abundance of important microbial metabolites such as bromobutyrate and acetate (Belizario and Faintuch, 2018). Bromobutyrate can affect cell metabolism by inhibiting the synthesis of fatty acids and can also promote the utilization of glucose. Acetate can regulate the acid–base balance, maintain intracellular environmental stability, and play a role in biochemical reactions such as the tricarboxylic acid cycle. Alterations in microbial composition and metabolic function have been documented to be related to the pathogenesis of GDM (Wang et al., 2020). It has been reported that the alteration of bacterial phyla seems to interfere with intestinal permeability, increasing the absorption of lipopolysaccharide (LPS), which can induce a chronic subclinical inflammatory process and obesity, leading to insulin resistance through the activation of TLR4 (Clare J Lee, 2020). Thus, therapy of GDM by regulating the gut microbiota and metabolome has emerged as a new therapeutic strategy.

As an active and major component in *Astragalus membranaceus* (Fisch) Bunge, AS IV exerts anti-diabetic (Lin et al., 2022), immunomodulatory (Zhong et al., 2022), anti-aging (Yao et al., 2023), anti-inflammatory (Li et al., 2023), and anti-oxidative neuroprotective effects (Ma et al., 2023). Additionally, several mechanisms have been proposed through which AS IV can exert its health effects by adjusting the gut microbiota composition (Hu et al., 2023). Moreover, AS IV may alter the abundance, diversity, and structure of the microbiota in the gut (Wu et al., 2023). However, the roles of AS IV and the mechanisms underlying its effects on gut microbiota and GDM development remain unclear. Therefore, we conducted this study to investigate whether AS IV exerts anti-GDM effects through the regulation of the microbiota composition and metabolites in the gut.

## 2 Materials and methods

### 2.1 Animal experiments

Female and male SD rats, weighing approximately 300 g, were provided by Pengyue Biological Company (Jinan, Shandong, China). All rats were maintained in well-ventilated plastic cages and under an individual 12/12-h dark/light condition. All our animal experiments were performed following the Guide for the Care and Use of Laboratory Animals by the National Institutes of Health. This study was approved by the Medical Research Ethics Committee of the Affiliated Hospital of Jining Medical University (ethics approval number 2022B031). Pentobarbital sodium (intraperitoneal injection, 150 mg/kg) was used for euthanasia. All endeavors were expended to reduce animal pain and discomfort. In this study, four groups of rats were set, namely, (1) control group, (2) GDM model group, (3) GDM model + AS IV group, and (4) AS IV group. AS IV was provided by Yuanye Biomart (B20564) and dissolved in propylene glycol (3 g/mL) (Sigma) for storage. Before usage, the stored AS VI solution was diluted using sodium chloride to a suitable concentration.

Via gavage, 10 mg/kg of the AS IV solution was administered to the rats in the GDM model + AS IV and AS IV groups once a day from 6 days before pregnancy to delivery. Equal volumes of the propylene glycol-contained sodium chloride solution were given to the control and GDM rats through gavage throughout the study as negative controls. All rats in the four groups were individually mated with male rats at a ratio of 2:1 at 8 p.m. In the following morning, designated gestational day (GD) 0, mating was confirmed by the presence of a copulatory plug or sperm observed under a vaginal smear microscope. Female rats that successfully conceived in three successive days were placed in the same batch. The streptozotocin (STZ, 45 mg/kg) intraperitoneal injection was carried out for the construction of the rat GDM model (Tian et al., 2015; Caluwaerts et al., 2003; Ma et al., 2022). The rats in the GDM model + AS IV and GDM model groups were injected with STZ (45 mg/kg) at GDs 1–4. The control and AS IV rats were injected with a citrate buffer solution at GDs 1–4. After STZ injection, blood glucose >11.1 mmol/L was considered to indicate GDM. Blood and fecal samples were collected on GD 19. In addition, the reproductive outcomes (the malformation rate and the number of normal fetuses) of all the groups were statistically recorded.

### 2.2 Glucose tolerance test

Fasting blood glucose levels of all rats were determined at GDs 0, 4, 9, and 19 using a glucometer (Roche, United States) after 8 h of fasting. Additionally, the body weights of all rats were measured using a top-loading balance (Thermo Fisher Scientific, United States).

### 2.3 Insulin level detection

Rats on GDs 1, 9, and 19 were let to fast for 8 h before blood sampling. Then, the capillary tubes were employed for the collection of the blood samples from the tail vein on GDs 1, 9, and 19. Serum insulin levels in pregnant rats were determined using an ELISA assay (Elabscience, E-EL-R3034, Wuhan, China). The value of insulin level × fasting blood glucose level/22.5 was defined as the insulin resistance index.

### 2.4 Measurement of lipopolysaccharide

Blood was collected on day 19 of pregnancy. The plasma samples were diluted tenfold with the sample treatment solution. Lipopolysaccharide levels in all pregnant rats were measured using an ELISA assay (Enzyme-linked Biotechnology, ml058913, Shanghai, China). Absorbance was measured at 450 nm using a microplate reader system (TECAN Infinite M200, Switzerland) operating in the absorbance mode at 37°C.

### 2.5 Measurement of IL-6 and TNF- α in serum

IL-6 (BMS625) and TNF-α (KRC3011) in the supernatants were measured using an ELISA assay from eBioscience. A microplate reader (TECAN Infinite M200) was employed for the measurement of the absorbance at 450 nm.

### 2.6 DNA extraction, PCR amplification, and sequencing of 16S rRNA

After collection, the feces of the rats were stored at −80°C immediately until use. A QIAamp Fast DNA Stool Mini Kit (QIAGEN, 51604, Germany) was used for the extraction of DNA from fecal samples. The extracted DNA was preserved at −80°C until further use. The 806R and 341F primers containing barcodes were employed to amplify the V4–V3 variable region of the 16S rRNA gene using DNA extracted from rat feces. The PCR amplification procedure was carried out using the Phusion High-Fidelity PCR Master Mix (Thermo Fisher Scientific, F548L, Sunnyvale, CA, United States). After mixing with a 1:1 ratio, the PCR products were purified with a QIAGEN Gel Extraction Kit (QIAGEN, 28706, Hilden, Germany). A TruSeq DNA PCR-Free Sample Preparation Kit (Illumina, FC-121-3001, United States) was employed for the preparation of the library for sequencing, and then, a Qubit 3.0 Fluorometer (Thermo Scientific, United States) and an Agilent Bioanalyzer 2100 system were used for the evaluation of the library. Finally, an Illumina HiSeq 2500 Platform (Novogene Bioinformatics Technology) was employed for the sequencing of the prepared library and generation of the 250-bp paired-end reads.

### 2.7 Analysis of fecal extracts using untargeted LC-MS

Fresh fecal samples from all groups were collected in 1.5-mL centrifuge tubes and then stored at −80°C until they were needed. Fecal samples (50 mg) from all groups were extracted, and proteins were sedimented with 800 μL of prechilled methanol containing 10 μL of DL-*o*-chlorophenylalanine (2.9 mg/mL, Yuanye Biomart, B25643, Shanghai, China). The samples were homogenized at 65 KHz for 100 s. After homogenization, the mixture was shaken for 5 min, followed by centrifugation at 12,000 g for 10 min at 4°C. Then, for further analysis, 200 μL of the supernatant was collected. Finally, an UltiMate 3000 LC Analyzer with a Hypergod C18 Column (100 × 4.6 mm 3 μm) was used for the LC-MS analysis using 60 μL of the supernatant. The settings for the LC-MS analysis were set as follows: automatic injector temperature, 4°C; mobile phase B, acetonitrile + 0.1% formic acid; mobile phase A, water + 0.1% formic acid; injection volume, 4 mL; column temperature, 40°C; and flow rate, 0.3 mL/min.

Compound Discoverer 2.3 software was used for the further extraction and pre-processing of all metabolomic data, such as compound molecular weight, peak areas, retention time, and observations (samples). MetaboAnalyst 3.0 (Montreal, QC, Canada) was used to analyze the pathway enrichment of these metabolites using the Kyoto Encyclopedia of Genes and Genomes (KEGG, http://www.genome.jp/kegg) database. For the quantitation for each metabolite, peak picking, and peak alignment, from the UHPLC-MS/MS-generated raw data, Compound Discoverer 3.1 (Thermo Fisher Scientific) was applied. The major parameters were set as follows: minimum intensity, 100,000; signal intensity tolerance, 30%; actual mass tolerance, 5 ppm; signal/noise ratio, 3; and retention time tolerance, 0.2 min. R software, Python, and CentOS were applied for the statistical analyses. The metabolites were explained using the LIPID MAPS database (http://www.lipidmaps.org/), KEGG database, and HMDB (https://hmdb.ca/metabolites). metaX was applied for partial least squares discriminant analysis (PLS-DA) and principal component analysis (PCA). The differentially changed metabolites were defined as *p*-values < 0.05, VIP > 1, and FC ≤ 0.5 or fold change ≥ 2.

### 2.8 Statistical analysis

The values of the data are expressed as the mean ± SD. The data analysis was conducted using SPSS. Spearman’s correlation analysis was performed using the R package and used to determine the correlation between the metabolome (positive and negative ions) and the microbiome in the gut (the genus level). Cytokine production was analyzed using GraphPad Prism, and R software was applied for the visualization of the results. The variance (ANOVA), Wilcoxon’s test, or Student’s t-test was conducted for the comparisons between or among multiple quantitative groups. A *p*-value of <0.05 was considered statistically significant for the analysis. MD means the mean difference.

## 3 Results

### 3.1 AS IV alleviated the symptoms of gestational diabetes in rats

**FIGURE 1:**
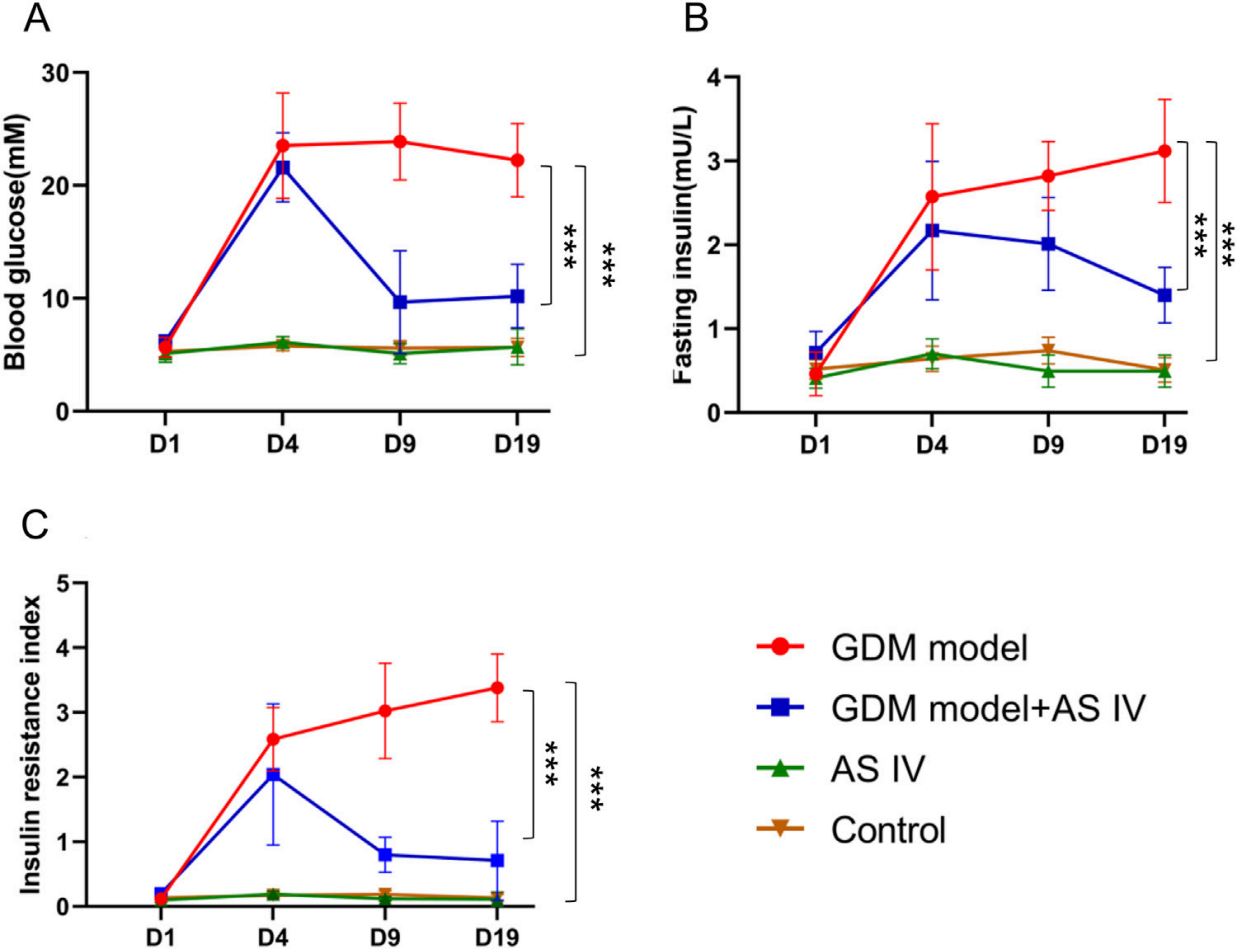
Effects of AS IV on fasting blood glucose and insulin levels in GDM model rats. Fasting blood glucose levels **(A)**, insulin levels **(B),** and the insulin resistance index **(C)** were determined on D1, D4, D9, and D19. All rats were fasted for 8 h before collecting the blood from the caudal vein. Data were shown as the mean ± SD. *n* = 6. *p < 0.05; **p < 0.01; and ***p-value ≤ 0.001.

The average duration of intervention of AS IV for the rats in the GDM model + AS IV group and AS IV group was 25 days. To assess the role of AS IV in GDM model rats, we determined insulin levels, fasting blood glucose levels, and the insulin resistance index of all rats. As opposed to rats from the control group, rats from the GDM group showed dramatically elevated fasting blood glucose levels (22.24 mM vs. 5.664 mM; MD = 16.57; *p* < 0.0001), insulin levels (3.120 mU/L vs. 0.508 mU/L; MD = 2.612; *p* < 0.0001), and the insulin resistance index (3.379 vs. 0.1309; MD = 3.248; *p* < 0.0001) (Figures 1A–C). In contrast, the elevated levels of insulin (1.399 mU/L vs. 3.120 mU/L; MD = 3.248;-1.720; *p* = 0.0001) and fasting blood glucose level (10.20 mM vs. 22.24 mM; MD = −12.04; *p* = 0.0003) in GDM rats on GD 19 were markedly decreased by the administration of AS IV (Figures 1A, B). In comparison to those non-treated GDM rats, the AS IV-treated GDM rats exhibited a dramatically reduced insulin resistance index (1.063 vs. 3.379; MD = −2.316; *p* = 0.0001) (Figure 1C). These results suggest that AS IV could effectively attenuate GDM symptoms in rats. In addition, on GD19 of pregnancy, compared with the control group, body weight (236.7 g vs. 283.7 g; MD = −46.99; *p* < 0.0001), water intake (351.6 mL/kg/day vs. 179.3 mL/kg/day; MD = 172.4; *p* < 0.0001), and feed intake (151.6 g/kg/day vs. 78.43 g/kg/day; MD = 73.12; *p* < 0.0001) were all significantly higher in the GDM model (Supplementary Figure S2). Compared with the GDM model, body weight (261.7 g vs. 236.7 g; MD = 25.04; *p* = 0.0006), water intake (157.9 mL/kg/day vs. 351.6 mL/kg/day; MD = 172.4; *p* < 0.0001), and feed intake (68 g/kg/day vs. 151.6 g/kg/day; MD = −83.55; *p* < 0.0001) were all significantly lower in the GDM model + AS IV group (Supplementary Figure S2).

### 3.2 AS IV treatment reduced lipopolysaccharide levels of the GDM model

**FIGURE 2:**
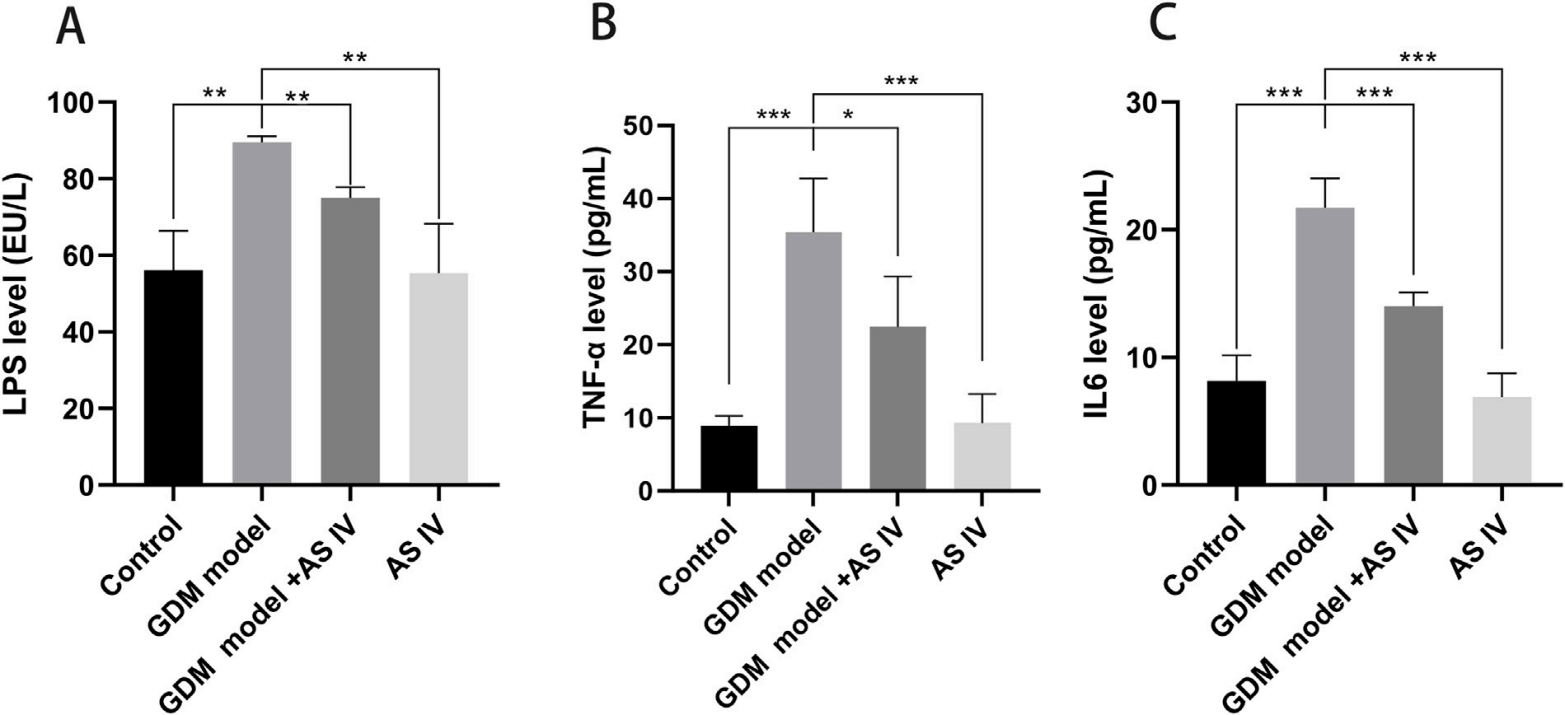
Effects of AS IV on the levels of LPS, TNF-α, and IL6 in GDM model rats. The levels of LPS **(A)**, TNF-α **(B),** and IL6 **(C)** were measured using an ELISA assay. A two-way analysis of variance was conducted with a Bonferroni *post hoc* multiple comparison test. Data were shown as the mean ± SD. *n* = 6. *p < 0.05; **p < 0.01; and ***p-value ≤ 0.001.

When compared to the control group of rats, the GDM rats exhibited an obviously high lipopolysaccharide level (88.28 EU/L vs. 56.10 EU/L; *p* = 0.0011). In contrast to the non-treated GDM rats, the AS IV-treated GDM rats exhibited drastic reduction in lipopolysaccharide levels (76.26 EU/L vs. 88.28EU/L; *p* = 0.0024) (Figure 2A).

### 3.3 AS IV reduces the secretion of TNF-α and IL6 in GDM rats

In comparison with the control rats, the GDM rats exhibited elevated production of TNF-α (35.38 pg/mL vs. 8.895 pg/mL; *p* = 0.0004) and IL-6 (21.716 pg/mL vs. 8.187 pg/mL, *p* = 0.0001). These elevated productions of IL-6 (14.00 pg/mL vs. 21.72 pg/mL; *p* = 0.0009) and TNF-α (22.44 vs. 35.38; *p* = 0.0432) were effectively restored by the administration of AS IV in GDM rats (Figures 2B, C).

### 3.4 AS IV improves the reproductive outcome of GDM rats

**FIGURE 3:**
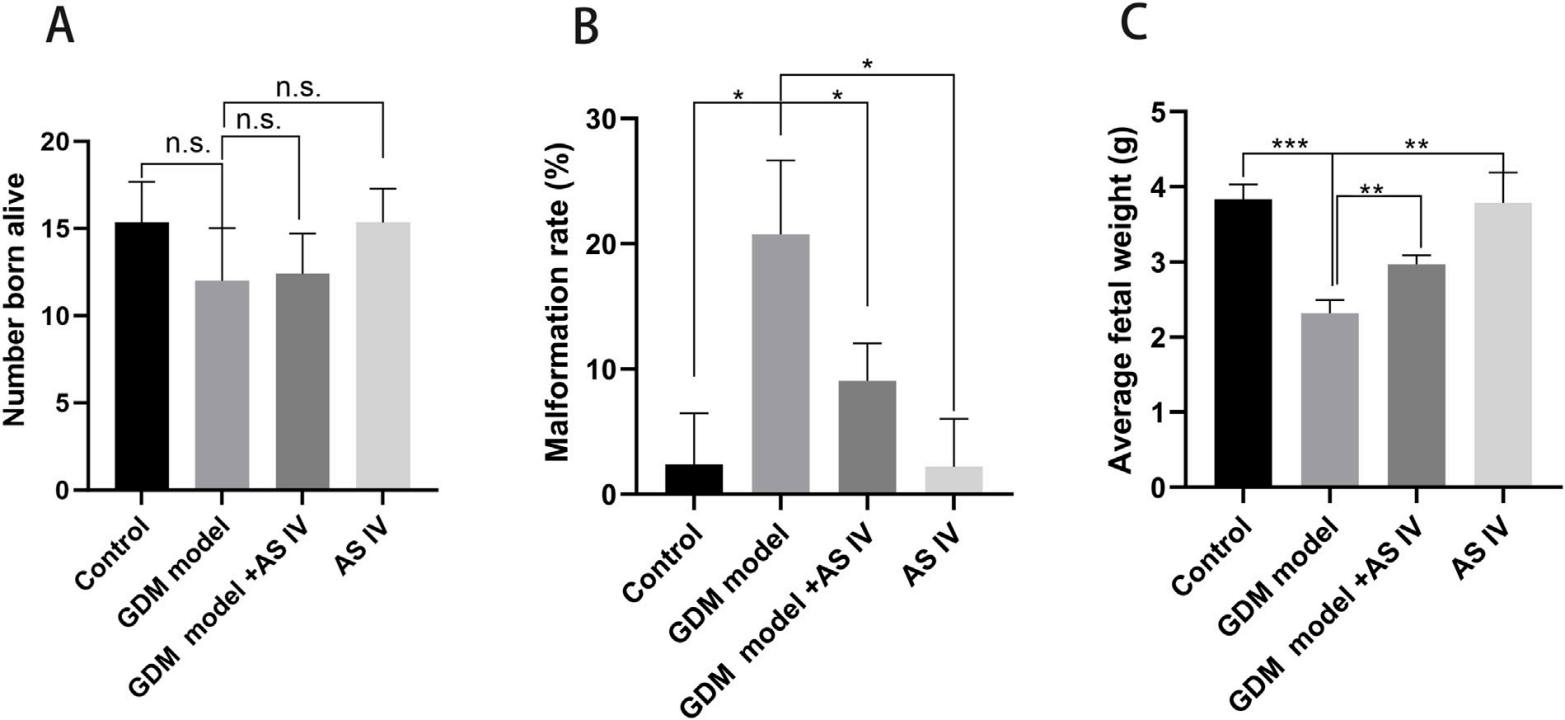
Effects of AS IV on the reproductive outcome of GDM model rats. The born-alive number **(A)**, the malformation rate **(B)**, and average fetal weight **(C)** were measured on the last day of gestation of all rats. A two-way analysis of variance was conducted with a Bonferroni *post hoc* multiple comparison test. Data were shown as the mean ± SD. *n* = 6. *p < 0.05; **p < 0.01; and ***p-value ≤ 0.001.

Reduced fetal survival, presented as a higher malformation rate and fewer normal fetuses, is one of the important adverse effects of GDM (Siemelink et al., 2002; Buckley et al., 2005). When compared to the rats of the control group, the GDM rats exhibited a high malformation rate (20.77 vs. 2,367; *p* = 0.0113) (Figure 3B). In addition, the average fetal weight of the GDM model group was significantly lower than that of the control group (2.320 vs. 3.835; *p* = 0.0001) (Figure 3C). Furthermore, we investigated whether AS IV treatment can improve fetal development in GDM model rats. The findings of our study revealed that compared to the vehicle-treated GDM rats, the AS IV-treated GDM rats showed a markedly decreased malformation rate (9.067 vs. 20.77; *p* = 0.0373) (Figure 3B). In addition, the average fetal weight of the AS IV-treated GDM rats was dramatically elevated compared with that of the non-treated GDM rats (2.970 vs. 2.320; *p* = 0.0020) (Figure 3C). However, the results showed that there was no statistical difference in the born-alive number among the four groups (*p* = 0.0588; *p* = 0.8144) (Figure 3A). These observations suggested that AS IV could enhance the reproductive outcome of GDM model rats.

### 3.5 Role of AS IV-mediated microbiotal diversity changes in GDM development

**FIGURE 4:**
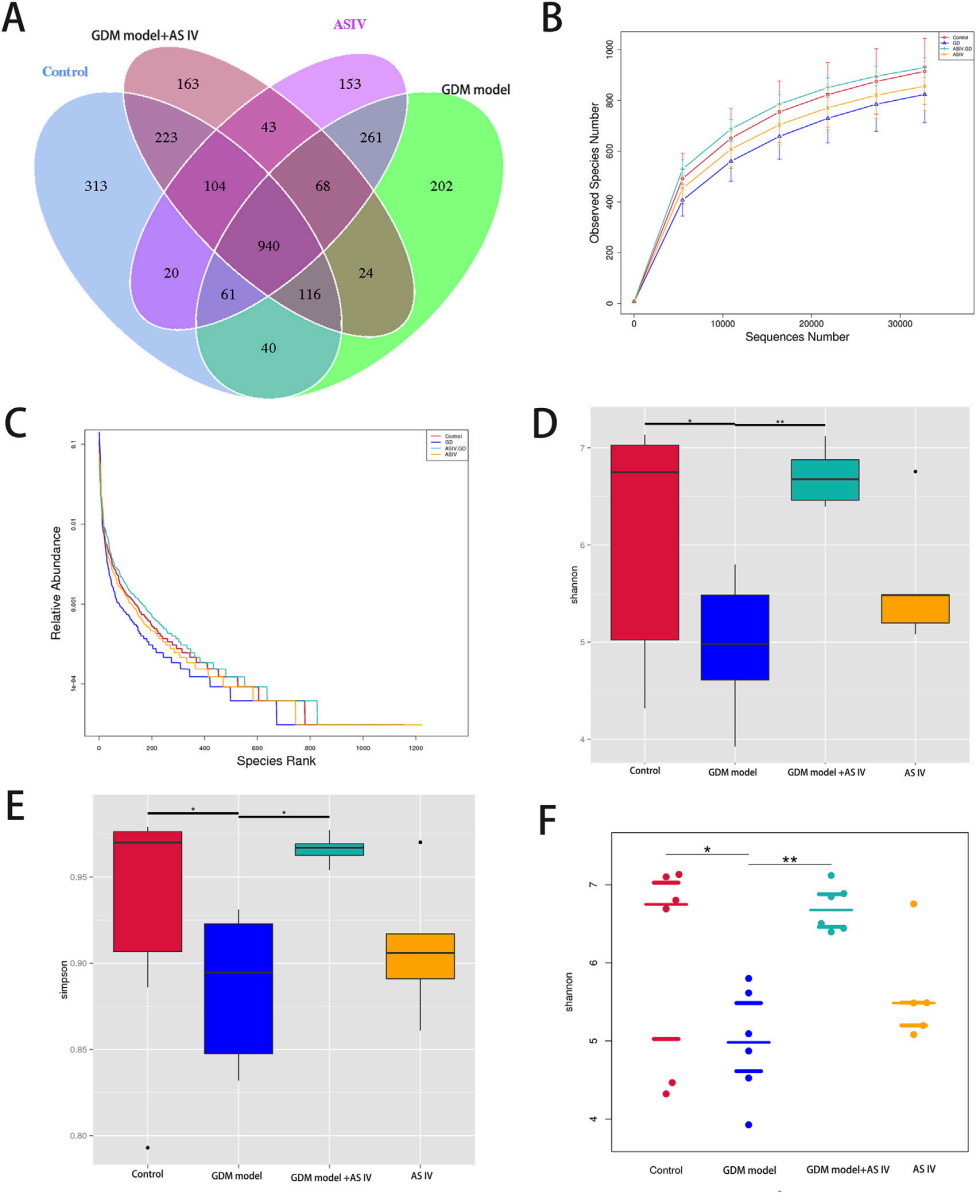
Effects of AS IV on the gut microbiome diversity of GDM model rats. **(A)** The Venn diagram shows the unique and shared OTUs of four groups (*n* = 6). **(B)** Rarefaction curves to estimate the richness of the gut microbiota in four groups. The vertical axis showed the number of OTUs expected after sampling the number of sequences or tags shown on the horizontal axis. **(C)** Rank abundance curves of bacterial OTUs derived from the four groups. Abundance-based coverage estimator, Shannon index **(D, F)**, and Simpson index **(E)**. A two-way analysis of variance was conducted with a Bonferroni *post hoc* multiple comparison test. Data were shown as the mean ± SD. *n* = 6. *p < 0.05; **p < 0.01; and ***p-value ≤ 0.001.

To illustrate the mechanism underlying the protective effect of AS IV treatment, we studied the impact of AS IV on the microbiota of the gut in a GDM model. To this end, we sequenced the V3–V4 region of 16S rRNA to evaluate the changes in the fecal microbiota of the GDM model with or without AS-IV treatment. A 97% similarity cutoff was applied for the determination of 1, 108, and 738 effective tags from 23 samples using QIIME. Fecal samples from the four groups showed 1,817 (control group), 1,812 (GDM model group), 1,681 (GDM model + AS IV group), and 1,650 (AS IV group) operational taxonomic units (OTUs) (Figure 4A). Among these, 940 bacteria were identified in all these 4 groups (Figure 4A). A stabilized OTU number was observed in the rarefaction curves when the number of reads >30,000 (Figure 4B). Additionally, similar species uniformity was found among these four groups by the abundance rank curves in the discovery set (Figure 4C). Then, in order to assess the abundance and diversity of the microbial community, alpha diversity analysis was conducted. The results showed that the GDM rats had a lower diversity (Simpson’s diversity index, Shannon diversity index, and Beeswarm) than the control rats (Figures 4D, E). AS IV treatment significantly increased Simpson’s diversity index, which indicated that the diversity was significantly increased (Figures 4D–F).

### 3.6 Effects of AS IV on the microbiota composition and abundance in the gut in GDM rats

**FIGURE 5:**
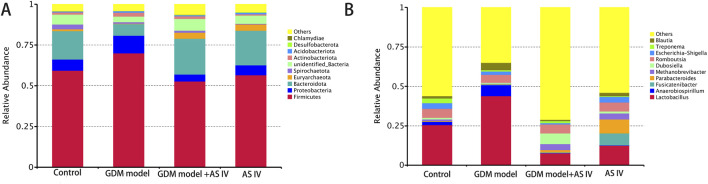
Gut microbiome composition and abundance analysis. **(A)** Histogram of the top 10 phyla in four groups; **(B)** Histogram of the top 10 genera in four groups.

The top 10 phyla in the microbiota of the gut in the discovery set are shown in Figure 5A. In the GDM group of rats, *Firmicutes* (phylum level) were the most abundant. In addition, AS IV significantly reduced the abundance of *Firmicutes* in GDM model rats. In comparison with the control rats, the GDM rats exhibited dramatically high *Proteobacteria* abundance. However, AS IV administration significantly reduced the abundance of *Proteobacteria* in the rats from the GDM group (Figure 5A). Furthermore, compared to those of control rats, we observed reduced abundance of *Bacteroidota*, *Euryarchaeota*, *Spirochaetota*, unidentified_bacteria, *Desulfobacterota*, and *Chlamydiae* in the rats with GDM. In addition, the abundance of *Bacteroidota*, *Euryarchaeota*, *Spirochaetota*, unidentified_bacteria, *Desulfobacterota*, and *Chlamydiae* was increased upon AS IV treatment (Figure 5A).

Figure 5B shows the top 10 microbiota genera of the gut in the discovery set. In comparison with the control rats, the GDM rats exhibited markedly increased levels of *Lactobacillus*, *Blautia*, and *Anaerobiospirillum* (the genus level). The GDM model + AS IV group was found to have a lower abundance of *Lactobacillus*, *Blautia*, and *Anaerobiospirillum* than the GDM group (Figure 5B). In addition, the GDM model group showed a lower abundance of *Methanobrevibacter*, *Treponema*, *Dubosiella*, and *Romboutsia* than the control group. Furthermore, the abundance of *Methanobrevibacter*, *Treponema*, *Dubosiella*, and *Romboutsia* was increased upon AS IV treatment (Figure 5B).

### 3.7 Effect of AS IV on the functional prediction of the microbiota in the gut in GDM rats

**FIGURE 6:**
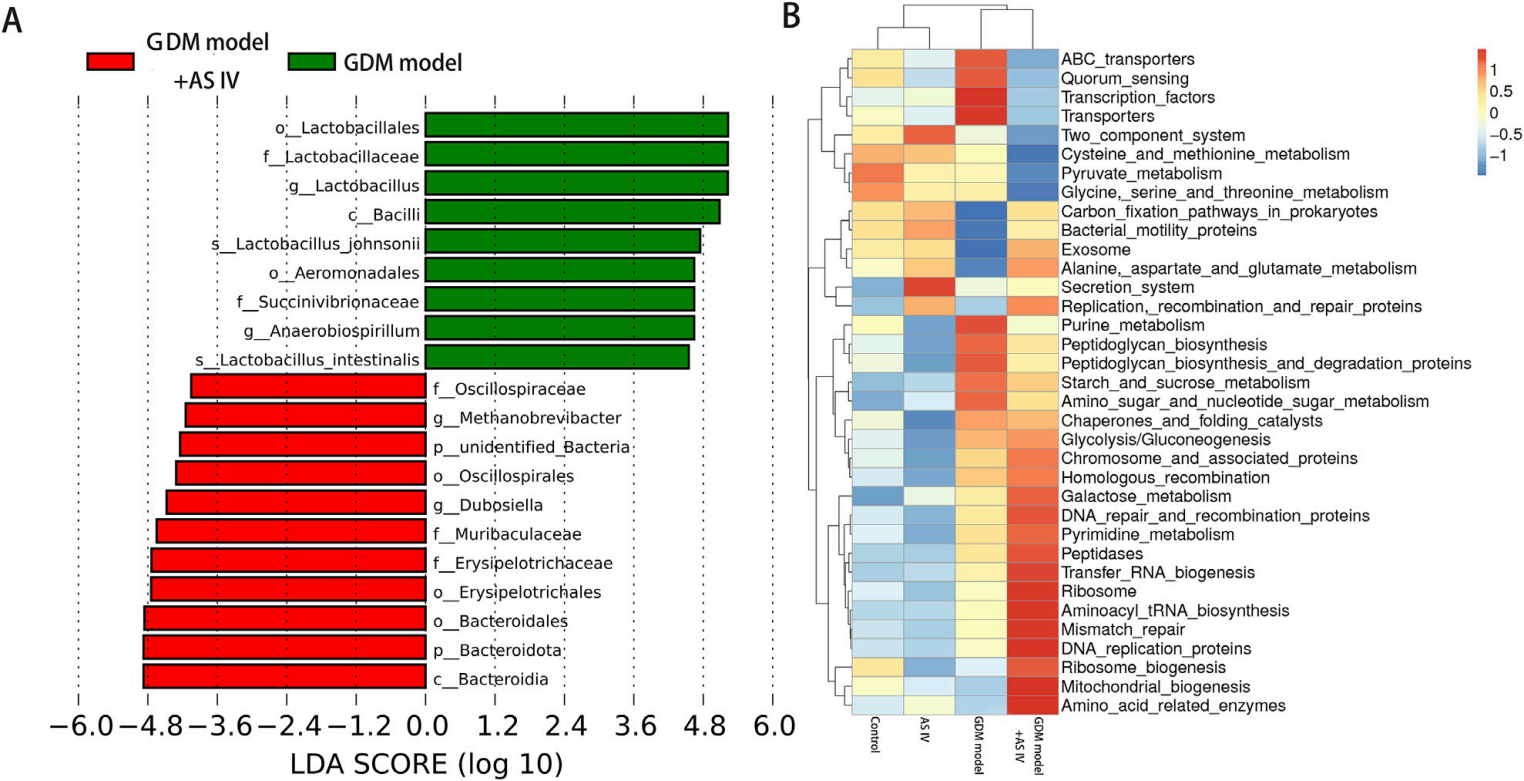
Analysis of significantly different microbes in the gut microbiome. **(A)** Significantly different microbes; LDA score means the linear discriminant analysis score; **(B)** heat map of Spearman’s rank correlations between differential genera (level 3).

In comparison with the GDM rats, the abundance of *Dubosiella* and *Methanobrevibacter* was significantly increased (Figure 6A). As shown in Figure 6B, the functional prediction of the microbiota in the gut was conducted using Tax4Fun software, and significant differences were observed in the functional abundance of 35 pathways at the KEGG pathway levels of 3. Thirteen metabolic pathways, including the metabolisms of cysteine, methionine, pyrimidine, amino sugar, nucleotide sugar, glycine, serine, threonine, galactose, pyruvate, purine, starch, and sucrose, were found (Figure 6B).

### 3.8 Effects of AS IV treatment on the characteristics of fecal metabolomics

**FIGURE 7:**
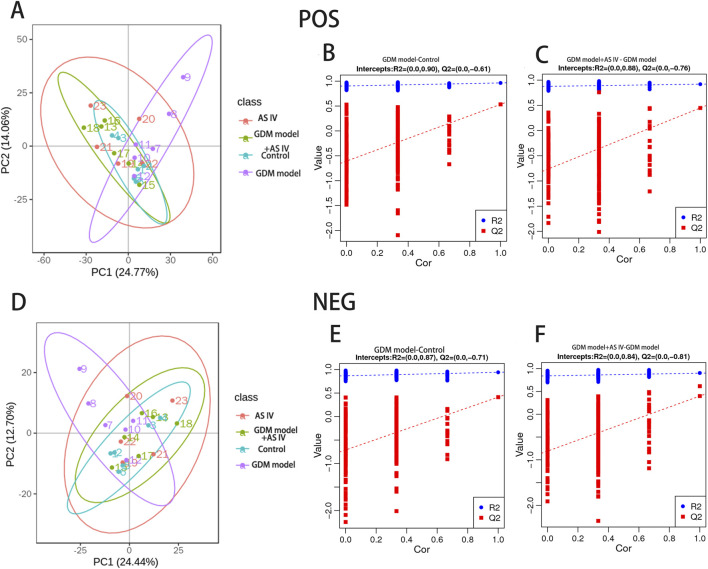
Multivariate statistical analysis of detected metabolites in positive **(A–C)** and negative ion modes **(D–F)**. PLS-DA scores: the abscissa represents the first principal component PC1. The ordinate represents the second principal component PC2. Replacement test diagram: R2 stands for model verification, and the Y matrix of the original classification and *N* times of different arrangements are linearly regressed with R2Y and Q2Y. The intercept values of regression line and *y*-axis are R2 and Q2, respectively, conditioned to determine whether the model is over-fitted.

**FIGURE 8:**
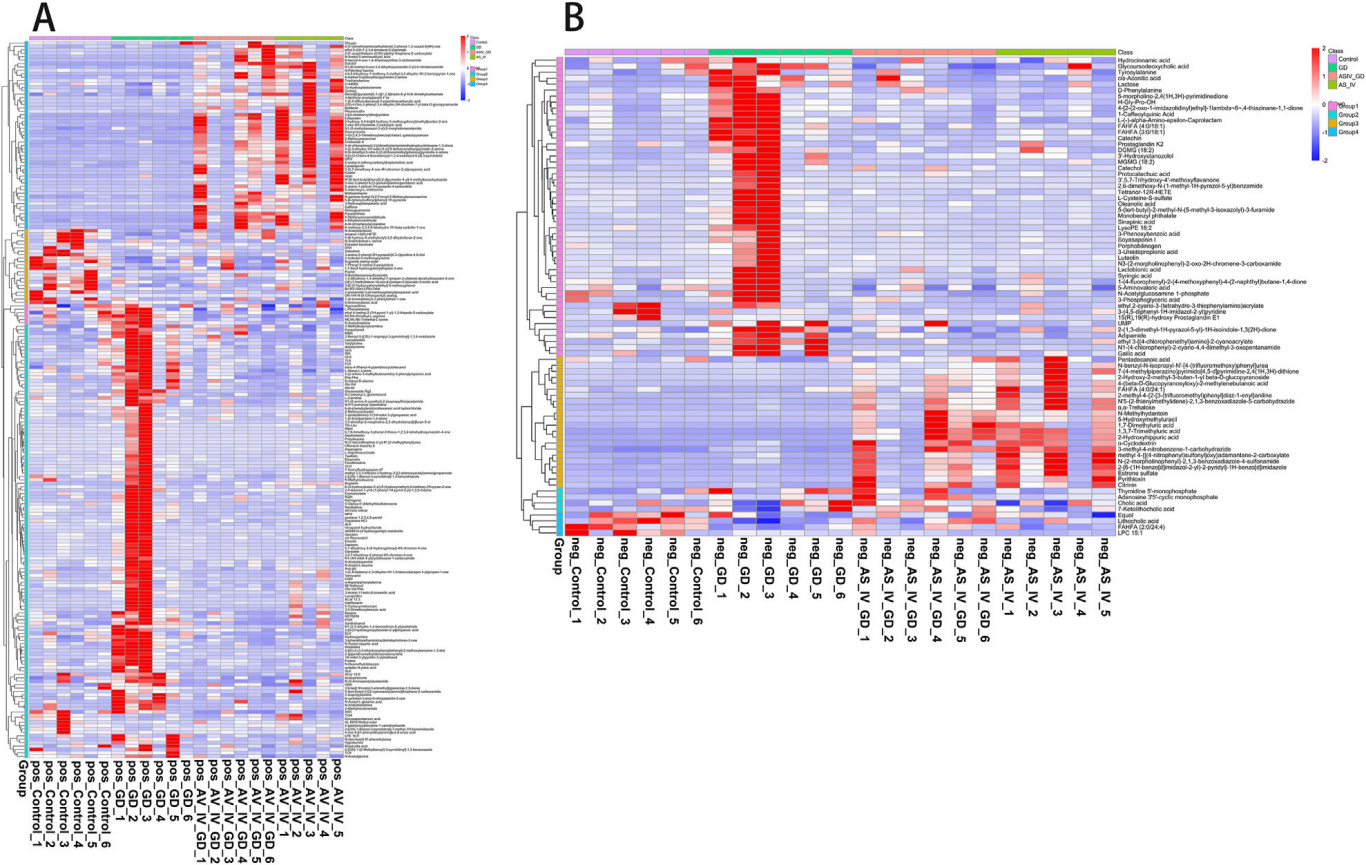
Statistics of identified metabolites: differences in metabolites of four groups in the positive ion model **(A)** and negative ion model **(B)**.

**FIGURE 9:**
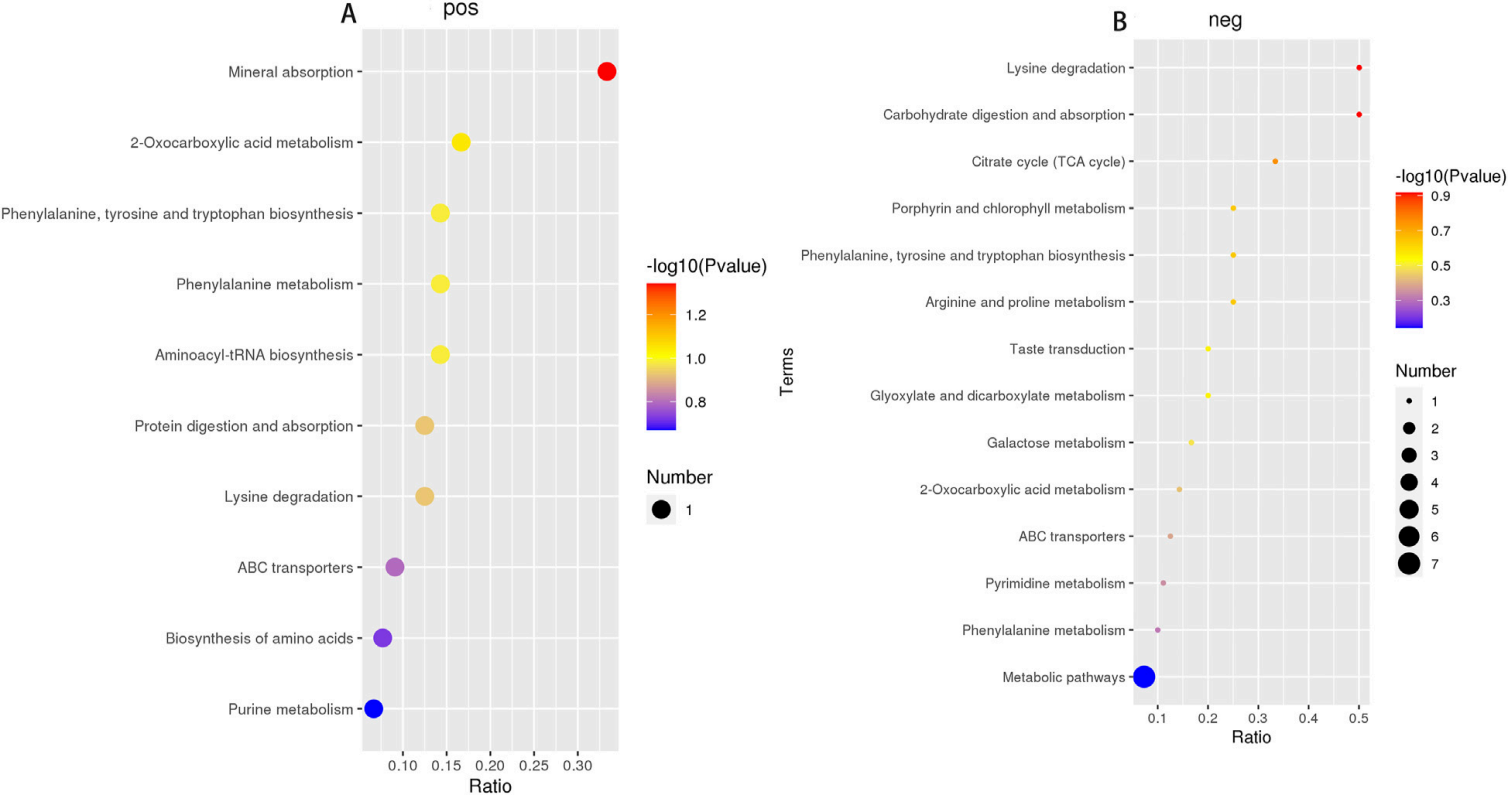
Metabolic pathway enrichment analysis of four groups in the positive ion model **(A)** and negative ion model **(B)**.

PLS-DA was carried out to determine the changes induced by AS IV treatment in GDM model rats. PLS-DA analysis was well-clustered. The analysis revealed metabolic differences in metabolites among the four groups (Figures 7A–F, the first row, first column, second row, and first column), suggesting that AS IV treatment inhibited biochemical changes in GDM model rats. Differentially enriched metabolites were identified among different groups in the negative and positive ion models (Supplementary Tables S1, S2). For example, in the positive model, compared to that of the control rats, the significantly high relative abundance of xanthohumol, d-alanyl-d-alanine, and valylproline was discovered in the GDM rats. Conversely, the increased abundance of these metabolites was effectively restored by the administration of AS IV. In addition, the GDM rats exhibited significantly reduced relative abundance of 2-isobutyl-3-methoxypyrazine, 1,7-bis(4-hydroxyphenyl) heptan-3-one, and N-acetyl-L-leucine compared to the control rats. However, this reduced abundance in GDM rats was dramatically restored by the administration of AS IV (Figure 8A). Furthermore, in the negative model, we also observed the relatively high abundance of lactose, tyrosylalanine, and glycodeoxycholic acid in the GDM rats compared to those in the control group (Figure 8B). Treatment with AS IV reduced the abundance of these metabolites. Moreover, the relative abundance of FAHFA (branched fatty acid esters of hydroxy fatty acids) (2:0/24:4), lithocholic acid, and LPC 15:1 was reduced in the GDM rats compared to those in the control group (Figure 8B). In addition, compared to that in the GDM rats, the relative abundance of FAHFA (2:0/24:4), lithocholic acid, and LPC 15:1 increased in the GDM model + AS IV group (Figure 8B). To further analyze the effects of AS IV treatment on the metabolism of GDM rats, KEGG pathway annotation and enrichment analyses were performed on the differential metabolites. The differential metabolites were primarily associated with metabolism, particularly 2-oxocarboxylic acid, mineral absorption, and other metabolic pathways (Figure 9).

### 3.9 Correlations of the differential metabolites with the microbiota in the gut

**FIGURE 10:**
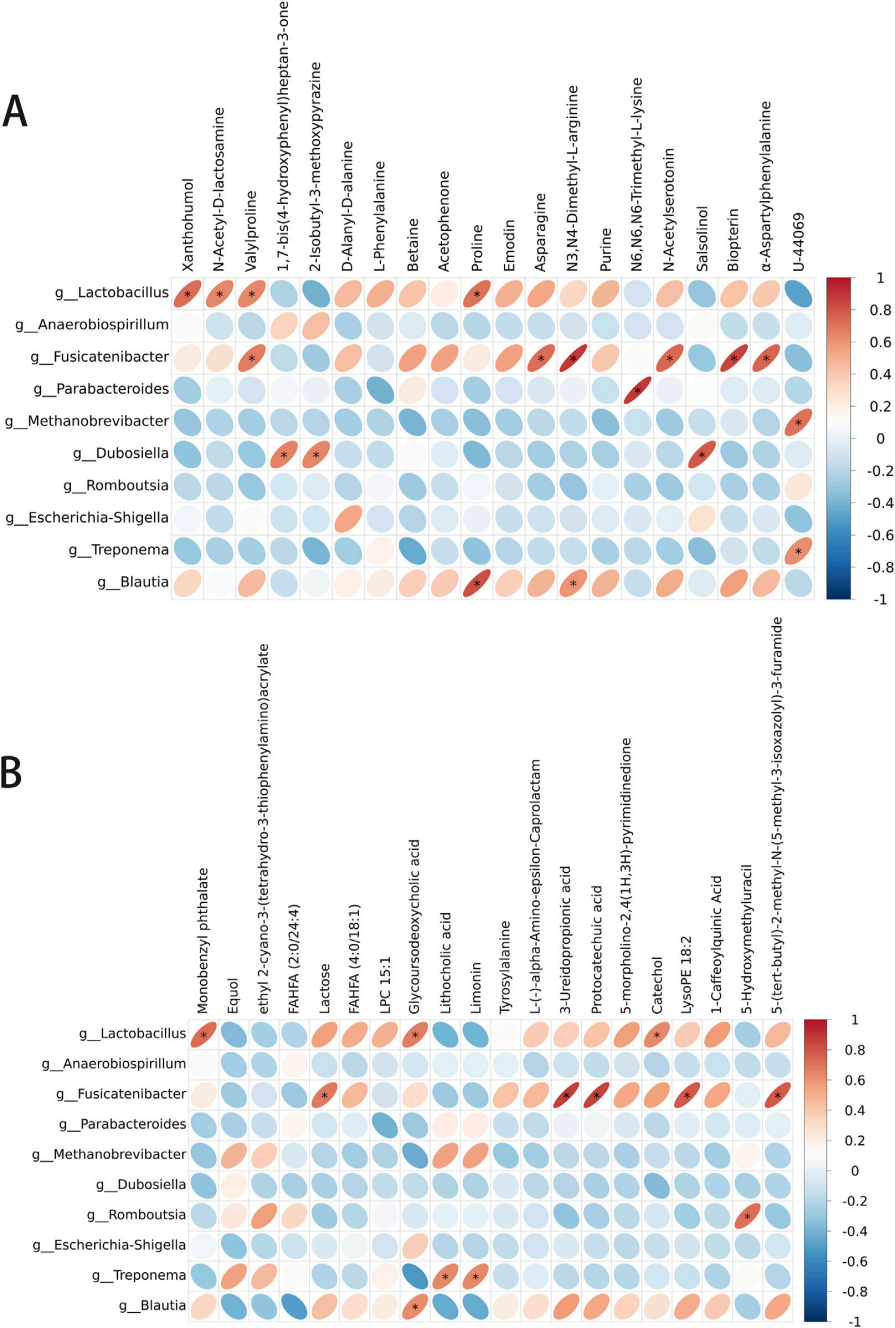
Spearman correlation analysis between the metabolite level and differential flora. **(A)** Correlation analysis between differential flora and positive ion metabolites. **(B)** Correlation analysis between differential flora and negative ion metabolites. *p-value < 0.05; **p-value < 0.01.

To determine whether the changes in the levels of gut metabolites were associated with gut microbiota composition, we assessed the correlation of the metabolites with the microbiota in the gut in the GDM model + AS IV and GDM model groups. The levels of 1,7-bis(4-hydroxyphenyl) heptan-3-one were positively correlated with *Dubosiella* in the positive model (Figure 10A). Meanwhile, we also observed a significantly positive association between *Lactobacillus* with the levels of N-Acetyl-D-lactosamine and xanthohumol and between the levels of proline with *Blautia* and *Lactobacillus* in the positive model (Figure 10A). Furthermore, lactose levels were positively related to *Fusicatenibacter* in the negative model (Figure 10B). Lithocholic acid levels were positively correlated with *Treponema* (Figure 10B). The levels of glycodeoxycholic acid were positively related to *Blautia* and *Lactobacillus* in the negative model (Figure 10B). These results are supported by the observations from the presence of metabolites and taxa enrichment.

## 4 Discussion

GDM is correlated with adverse maternal and neonatal outcomes. Although many clinical and basic studies have been performed on diabetes, very few have specifically targeted GDM, which adopts the dietary intervention approach (Landon et al., 2009). Hence, exploring and identifying the natural compounds for GDM treatment safely and effectively is urgently needed. Because of its well-known effects in promoting generation, draining abscesses, reinforcing diuretic, benefitting the skin, and tonifying vital energy, *A. membranaceus* (Fisch.) Bunge is considered a commonly employed Chinese medicinal herb. Our study showed that AS IV could relieve GDM by altering the bacterial community composition and function. Notably, AS IV treatment alleviated the symptoms of gestational diabetes in rats in the present study.

During the late trimester of pregnancy, a pregnant person with gestational diabetes exhibits low glucose uptake rates, decreased insulin secretion, and high insulin resistance (Pinto et al., 2023). The imbalance of insulin in the mother, caused by an aggravated insulin-resistant state and reduced insulin secretion, could induce glucose intolerance and lead to the development of GDM (Di Cianni et al., 2003). In this study, the administration using AS IV could effectively decrease the glucose level in the blood, reduce the level of insulin, and inhibit glucose intolerance and insulin resistance in GDM model rats. Low-grade maternal inflammation is a hallmark of pregnancy complications associated with GDM (Gauster M. et al., 2012). In addition, high LPS levels are associated with GDM (Kuang et al., 2017). AS IV treatment reduced the serum levels of lipopolysaccharide, which is an important serological index for monitoring abnormal glucose metabolism (Tola A Faraj and Erridge, 2017), TNF-α, and IL6 in the GDM model. Our findings suggest that AS IV may be an alternative and effective approach to reducing blood glucose levels in pregnant women with GDM. Another important finding of our study is that AS IV treatment improved fetal outcomes in pregnant rats with GDM. One of the major adverse symptoms of GDM is fetal maldevelopment (Gajagowni et al., 2022). AS IV significantly increased the average fetal weight and reduced the malformation rate compared to the GDM rats, suggesting an improved reproductive outcome in GDM rats after AS IV administration.

As an important modulator, diet is significantly connected to the diversity and composition of the microbiota in the gut and affects the processes of physiology and metabolism, thus playing a vital role in host health (Guan and Liu, 2023). To assess the effects of AS IV on GDM, the genes of 16S rRNA in the fecal microorganisms were explored. The stability and health of the host can be evaluated by the diversity of the microbiota (Clarke et al., 2014). According to bioinformatics analyses of the discovery set, AS IV inhibited the decrease in richness (alpha diversity) in GDM model rats, suggesting that AS IV could modify fecal microbiota alpha diversity. *Proteobacteria, Firmicutes, Actinobacteria,* and *Bacteroidetes* are the major phyla constituting the microbiota of the gut in humans and rats (Haange et al., 2012). Consistent with these studies, our results also showed that the aforementioned bacteria are the most abundant gut microorganisms in rats.

Increased maternal insulin resistance in GDM patients in late pregnancy, in combination with impaired β-cell function, leads to glucose intolerance (Mittendorfer et al., 2023). Moreover, the resistance of insulin is closely connected to the enhanced abundance of *Firmicutes* and *Bacteroidete*s (Song et al., 2016). It has been reported that levels of glucagon-like peptide 1 (GLP-1) decreased with the increased abundance of *Firmicutes* and *Bacteroidetes*, which could be the cause of the change in insulin resistance (Greenhill, 2015). In this study, AS IV treatment prevented an increase in insulin resistance in the GDM model rats. In addition, compared to the rats of the control group, the GDM rats exhibited significantly high *Actinobacteria, Proteobacteria,* and *Firmicutes* abundance and low *Bacteroidetes* abundance. However, the elevated *Actinobacteria, Proteobacteria,* and *Firmicutes* abundance was obviously suppressed by the administration of AS IV, suggesting AS IV may inhibit the reduction of GLP-1 by preventing changes in bacterial composition and distribution of *Firmicutes* and *Bacteroidetes*, ultimately achieving the effect of suppressing GDM. *Proteobacteria* can cause various types of inflammation and tissue damage (Jie Cai et al., 2022). Low-grade maternal inflammation is a hallmark of pregnancy complications associated with GDM (Gauster G. D. M. et al., 2012). Therefore, given the significant AS IV-induced reduction in the abundance of *Proteobacteria*, AS IV may be a useful strategy for preventing and alleviating GDM by reducing the proliferation of pathogenic bacteria.

Our results observed a high abundance of *Blautia* and *Anaerobiospirillum* in the GDM rats compared to that of control rats at the genus level. Patients with GDM show increased levels of *Blautia* and *Anaerobiospirillum* (Wu et al., 2021). In addition, *Blautia* is enriched in the gut microbiota of GDM patients (Ye et al., 2019). An increase in *Blautia* may contribute significantly to GDM development (Min Yan et al., 2023). *Anaerobiospirillum succiniciproducens*, which is a maleficent bacterium, is a Gram-negative anaerobic rod. The administration of AS IV markedly decreased the abundance of *Lactobacillus*, *Blautia*, and *Anaerobiospirillum*, implying that AS IV may inhibit GDM development by decreasing the abundance of *Blautia* and *Anaerobiospirillum. Lactobacillus* is a beneficial bacterium that has been reported to reduce GDM incidence (Si et al., 2019; Zhang et al., 2021; Reyes-Munoz et al., 2020). Interestingly, compared to other groups of rats, the GDM rats showed a significantly high abundance of *Lactobacillus*. Probiotic microorganisms have long been considered innocuous, but recent data suggest that *Lactobacillus* may affect certain infections, inflammation, allergies, or antibiotic gene transfer (Mantegazza et al., 2018). Thus, we speculate that excessive *Lactobacillus* may affect certain inflammatory gene transfers as gestational diabetes presents as a state of inflammation. Moreover, compared to the control rats, the GDM rats exhibited an extremely decreased abundance of *Methanobrevibacter*, *Dubosiella*, and *Romboutsia*. However, this reduced abundance of *Methanobrevibacter, Dubosiella, and Romboutsia* was effectively enhanced by the treatment of AS IV. Furthermore, the finding showed significant differences in the functional abundance of 35 KEGG pathways, including glutamate and alanine aspartate metabolism and nucleotide and amino sugar metabolism. AS IV may exerts its anti-GDM effects through the differential metabolites and the pathways related to the metabolism of these metabolites. As a progenitor molecule, glutamine could effectively induce the production of insulin (Wurtz et al., 2012). Therefore, the metabolism of glutamine/glutamic acid may be disrupted by the changes in the metabolic pathways associated with GDM, thereby affecting the e3n energy balance. AS IV treatment may relieve GDM by modulating the intestinal flora and further changing the activities of metabolic pathways. In summary, AS IV treatment may prevent or treat GDM by reconstructing the homeostasis of the gut microbiota.

Our study determined the effects of AS IV on gut metabolic profiles using LC-MS. Limonin is known for its diverse range of pharmacological actions, including its potent anti-inflammatory, anti-cancer, anti-oxidant, and analgesic properties (Shin et al., 2020). In this study, a prominent decrease in limonin levels was observed in the GDM model. In contrast, in comparison with the GDM and control rats, AS IV-treated GDM rats showed significantly increased limonin. Our result suggested that AS IV may inhibit GDM by modifying the level of limonin and inhibiting the inflammatory response. Equol can ameliorate insulin secretion failure via Chrebp/Txnip signaling by modulating the activity of the PKA/PP2A signal pathway (Chen et al., 2020). AS IV treatment inhibited the reduction in equol levels, suggesting that AS IV may inhibit GDM by modulating equol levels and improve insulin secretion failure through Chrebp/Txnip signaling by modulating PKA/PP2A signaling activity. Several genera of bacteria, which are closely involved in the host metabolism, were identified using correlational analyses. Proline was positively correlated with *Blautia* and *Lactobacillus.* Proline possesses pharmacologic action, including anti-inflammatory and anti-apoptosis effects (Kazberuk et al., 2020; Andrade et al., 2018). However, the exact mechanism and role of proline in the anti-GDM effects of AS IV remain unclear. Inflammation and oxidative stress in placental cells are the main pathogenic mechanisms of GDM (De Luccia et al., 2020). Therefore, we speculated that the imbalance of intestinal flora may lead to a change in the proline content, which leads to cell apoptosis and, ultimately, to the occurrence of GDM. Thus, we predicted that AS IV treatment might strengthen the interaction between gut microbiota and proline by regulating gut metabolite profiles. Valylproline was positively correlated with *Dubosiella* and *Fusicatenibacter*. Valylproline, a free radical scavenger, exerts anti-inflammatory effects (Dahiya and Singh, 2017). However, the exact mechanism and role of valylproline in the anti-GDM effects of AS IV are unclear, and we inferred that the dysregulation of the intestinal flora could lead to a decrease in the valylproline content and increase the inflammatory response, thereby contributing to GDM occurrence. In addition, AS IV treatment may relieve GDM by modulating the gut microbiota and further changing the valylproline content. To further clarify the effects of gut dysbiosis and its metabolites on GDM pathogenesis, further studies are needed. Hence, by regulating gut metabolite profiles, AS IV supplementation may strengthen the association between the gut microbiota and metabolites.

The strength of this study lies in its discovery that AS IV can prevent the development of gestational diabetes. Moreover, our research is the first to demonstrate that supplementation with AS IV may modify gut microbiota composition and metabolites, thereby preventing GDM. However, our study also has several limitations. First, the sample size is small. In future, larger sample sizes are needed for further validation before conducting clinical trials. Second, due to the limitation of 16S sequencing, which cannot to detect species-level differences compared to shotgun sequencing, the deeper mechanisms linking the microbiota and their metabolites remain unclear.

## 5 Conclusion

Our findings demonstrated that GDM increased alterations in the microbiota and metabolome profiles and the pathogenic bacteria’s abundance. In contrast, AS IV treatment protected the rats from GDM by modulating the gut microbiota and metabolome profiles. AS IV treatment may relieve GDM by modulating the gut microbiota, which further changes the activities of valylproline, proline, and other metabolites, thus increasing anti-inflammatory effects and decreasing the cell apoptosis ratio, among other effects. Future studies should be performed to clarify the mechanisms underlying the connection between the microbiota and their metabolites in the gut.

## Data Availability

Publicly available datasets were analyzed in this study. The 16S rRNA sequencing data presented in the study have been submitted to NCBI (SUB11332010); the LC-MS data presented in the study are deposited in the MetaboLights (study identifier: MTBLS4677).

## Supplementary material

SUPPLEMENTARY TABLE S1 Statistics of identified metabolites: identified differential fecal metabolites among four groups in the positive ion model.

SUPPLEMENTARY TABLE S2 Statistics of identified metabolites: identified differential fecal metabolites among four groups in the negative ion model.

SUPPLEMENTARY FIGURE S1 Flowchart summarizing the flow of intervention of this study.

SUPPLEMENTARY FIGURE S2 Effects of AS IV on the body weight, water intake, and feed intake of GDM model rats. The body weight **(A)**, water intake **(B)**, and feed intake **(C)** were measured of all rats. **p*-value < 0.05; ***p*-value < 0.01; and ****p*-value ≤ 0.001.

## References

[B1] Aldars-Garcia L. Chaparro M. Gisbert J. P. (2021). Systematic review: the gut microbiome and its potential clinical application in inflammatory bowel disease. Microorganisms 9, 977. 10.3390/microorganisms9050977 33946482 PMC8147118

[B2] Andrade V. S. Rojas D. B. de Andrade R. B. Kim T. D. H. Vizuete A. F. Zanatta A. (2018). A possible anti-inflammatory effect of proline in the brain cortex and cerebellum of rats. Mol. Neurobiol. 55, 4068–4077. 10.1007/s12035-017-0626-z 28585188

[B3] Belizario J. E. Faintuch J. (2018). Microbiome and gut dysbiosis. Exp. Suppl. 109, 459–476. 10.1007/978-3-319-74932-7_13 30535609

[B4] Buckley A. J. Keseru B. Briody J. Thompson M. Ozanne S. E. Thompson C. H. (2005). Altered body composition and metabolism in the male offspring of high fat-fed rats. Metabolism 54, 500–507. 10.1016/j.metabol.2004.11.003 15798958

[B5] Caluwaerts S. Holemans K. van Bree R. Verhaeghe J. Van Assche F. A. (2003). Is low-dose streptozotocin in rats an adequate model for gestational diabetes mellitus? J. Soc. Gynecol. Invest 10, 216–221. 10.1016/s1071-5576(03)00044-3 12759150

[B6] Chen K. Lang H. Wang L. Liu K. Zhou Y. Mi M. (2020). S-Equol ameliorates insulin secretion failure through Chrebp/Txnip signaling via modulating PKA/PP2A activities. Nutr. Metab. (Lond) 17, 7. 10.1186/s12986-020-0426-8 31956333 PMC6961363

[B7] Clare J Lee C. L. S. (2020). Nisa Maruthur Gut microbiome and its role in obesity and insulin resistance. Ann. N. Y. Acad. Sci. 1, 37–52. 10.1111/nyas.14107 31087391

[B8] Clarke S. F. Murphy E. F. O'Sullivan O. Lucey A. J. Humphreys M. Hogan A. (2014). Exercise and associated dietary extremes impact on gut microbial diversity. Gut 63, 1913–1920. 10.1136/gutjnl-2013-306541 25021423

[B9] Dahiya R. Singh S. (2017). Synthesis, characterization, and biological activity studies on fanlizhicyclopeptide A. Iran. J. Pharm. Res. 16, 1176–1184. 29201105 PMC5610772

[B10] De Luccia T. P. B. Pendeloski K. P. T. Ono E. Mattar R. Pares D. B. S. Yazaki Sun S. (2020). Unveiling the pathophysiology of gestational diabetes: studies on local and peripheral immune cells. Scand. J. Immunol. 91, e12860. 10.1111/sji.12860 31849072

[B11] Di Cianni G. Miccoli R. Volpe L. Lencioni C. Del Prato S. (2003). Intermediate metabolism in normal pregnancy and in gestational diabetes. Diabetes Metab. Res. Rev. 19, 259–270. 10.1002/dmrr.390 12879403

[B12] Ferrocino I. Ponzo V. Gambino R. Zarovska A. Leone F. Monzeglio C. (2018). Changes in the gut microbiota composition during pregnancy in patients with gestational diabetes mellitus (GDM). Sci. Rep. 8, 12216. 10.1038/s41598-018-30735-9 30111822 PMC6093919

[B13] Gajagowni S. Nair P. Bapat A. C. Vachharajani A. J. (2022). Diabetic embryopathies. Neoreviews 23, e677–e688. 10.1542/neo.23-10-e677 36180736

[B14] Gauster G. D. M. Tötsch M. Hiden U. (2012b). The placenta and gestational diabetes mellitus. Curr. Diabetes Rep. 12, 16–23. 10.1007/s11892-011-0244-5 22102097

[B15] Gauster M. Desoye G. Totsch M. Hiden U. (2012a). The placenta and gestational diabetes mellitus. Curr. Diab Rep. 12, 16–23. 10.1007/s11892-011-0244-5 22102097

[B16] Gray S. G. McGuire T. M. Cohen N. Little P. J. (2017). The emerging role of metformin in gestational diabetes mellitus. Diabetes Obes. Metab. 19, 765–772. 10.1111/dom.12893 28127850

[B17] Greenhill C. (2015). Gut microbiota: Firmicutes and Bacteroidetes involved in insulin resistance by mediating levels of glucagon-like peptide 1. Nat. Rev. Endocrinol. 5, 254. 10.1038/nrendo.2015.40 25781856

[B18] Guan L. Liu R. (2023). The role of diet and gut microbiota interactions in metabolic homeostasis. Adv. Biol. (Weinh) 7, e2300100. 10.1002/adbi.202300100 37142556

[B19] Haange S. B. Oberbach A. Schlichting N. Hugenholtz F. Smidt H. von Bergen M. (2012). Metaproteome analysis and molecular genetics of rat intestinal microbiota reveals section and localization resolved species distribution and enzymatic functionalities. J. Proteome Res. 11, 5406–5417. 10.1021/pr3006364 23016992

[B20] Hu E. Li Z. Li T. Yang X. Ding R. Jiang H. (2023). A novel microbial and hepatic biotransformation-integrated network pharmacology strategy explores the therapeutic mechanisms of bioactive herbal products in neurological diseases: the effects of Astragaloside IV on intracerebral hemorrhage as an example. Chin. Med. 18, 40. 10.1186/s13020-023-00745-5 37069580 PMC10108474

[B21] Jardon K. M. Canfora E. E. Goossens G. H. Blaak E. E. (2022). Dietary macronutrients and the gut microbiome: a precision nutrition approach to improve cardiometabolic health. Gut 71, 1214–1226. 10.1136/gutjnl-2020-323715 35135841 PMC9120404

[B22] Jie Cai L. S. Gonzalez F. J. Gonzalez F. J. (2022). Gut microbiota-derived bile acids in intestinal immunity, inflammation, and tumorigenesis. Cell Host Microbe 30, 289–300. 10.1016/j.chom.2022.02.004 35271802 PMC8923532

[B23] Kazberuk A. Zareba I. Palka J. Surazynski A. (2020). A novel plausible mechanism of NSAIDs-induced apoptosis in cancer cells: the implication of proline oxidase and peroxisome proliferator-activated receptor. Pharmacol. Rep. 72, 1152–1160. 10.1007/s43440-020-00140-z 32710395 PMC7550302

[B24] Kelly J. R. Borre Y. C O. B. Patterson E. El Aidy S. Deane J. (2016). Transferring the blues: depression-associated gut microbiota induces neurobehavioural changes in the rat. J. Psychiatr. Res. 82, 109–118. 10.1016/j.jpsychires.2016.07.019 27491067

[B25] Kuang Y. S. Lu J. H. Li S. H. Li J. H. Yuan M. Y. He J. R. (2017). Connections between the human gut microbiome and gestational diabetes mellitus. Gigascience 6, 1–12. 10.1093/gigascience/gix058 PMC5597849 28873967

[B26] Landon M. B. Spong C. Y. Thom E. Carpenter M. W. Ramin S. M. Casey B. (2009). A multicenter, randomized trial of treatment for mild gestational diabetes. N. Engl. J. Med. 361, 1339–1348. 10.1056/NEJMoa0902430 19797280 PMC2804874

[B27] Li G. Yin P. Chu S. Gao W. Cui S. Guo S. (2021). Correlation analysis between GDM and gut microbial composition in late pregnancy. J. Diabetes Res. 2021, 8892849. 10.1155/2021/8892849 33628840 PMC7889370

[B28] Li L. Zhang Y. Luo Y. Meng X. Pan G. Zhang H. (2023). The molecular basis of the anti-inflammatory property of astragaloside IV for the treatment of diabetes and its complications. Drug Des. Devel Ther. 17, 771–790. 10.2147/DDDT.S399423 PMC10013573 36925998

[B29] Lin Y. Xu Y. Zheng X. Zhang J. Liu J. Wu G. (2022). Astragaloside IV ameliorates streptozotocin induced pancreatic β-cell apoptosis and dysfunction through SIRT1/P53 and Akt/GSK3β/Nrf2 signaling pathways. Diabetes Metab. Syndr. Obes. 15, 131–140. 10.2147/DMSO.S347650 35046684 PMC8763261

[B30] Ma J. Chen T. Wang R. (2023). Astragaloside IV ameliorates cognitive impairment and protects oligodendrocytes from antioxidative stress via regulation of the SIRT1/Nrf2 signaling pathway. Neurochem. Int. 167, 105535. 10.1016/j.neuint.2023.105535 37209830

[B31] Ma Y. F. Xu S. Meng J. Li L. (2022). Protective effect of nimbolide against streptozotocin induced gestational diabetes mellitus in rats via alteration of inflammatory reaction, oxidative stress, and gut microbiota. Environ. Toxicol. 37, 1382–1393. 10.1002/tox.23491 35212444

[B32] Mahajan A. Donovan L. E. Vallee R. Yamamoto J. M. (2019). Evidenced-based nutrition for gestational diabetes mellitus. Curr. Diab Rep. 19, 94. 10.1007/s11892-019-1208-4 31473839

[B33] Mantegazza C. Molinari P. D'Auria E. Sonnino M. Morelli L. Zuccotti G. V. (2018). Probiotics and antibiotic-associated diarrhea in children: a review and new evidence on Lactobacillus rhamnosus GG during and after antibiotic treatment. Pharmacol. Res. 128, 63–72. 10.1016/j.phrs.2017.08.001 28827186

[B34] Min Yan X. G. Ji G. Huang R. Huang D. Li Z. Zhang D. (2023). Mechanismbased role of the intestinal microbiota in gestational diabetes mellitus: a systematic review and meta-analysis. Front. Immunol. 13, 1097853. 10.3389/fimmu.2022.1097853 36936475 PMC10020587

[B35] Mittendorfer B. Patterson B. W. Haire-Joshu D. Cahill A. G. Cade W. T. Stein R. I. (2023). Insulin sensitivity and β-cell function during early and late pregnancy in women with and without gestational diabetes mellitus. Diabetes Care 46, 2147–2154. 10.2337/dc22-1894 37262059 PMC10698210

[B36] Moloney R. D. Desbonnet L. Clarke G. Dinan T. G. Cryan J. F. (2014). The microbiome: stress, health and disease. Mamm. Genome 25, 49–74. 10.1007/s00335-013-9488-5 24281320

[B37] Pinto Y. Frishman S. Turjeman S. Eshel A. Nuriel-Ohayon M. Shrossel O. (2023). Gestational diabetes is driven by microbiota-induced inflammation months before diagnosis. Gut 72, 918–928. 10.1136/gutjnl-2022-328406 36627187 PMC10086485

[B38] Reyes-Munoz E. Sosa S. E. Y. Flores-Robles C. M. Arce-Sanchez L. Martinez-Cruz N. Garduno-Garcia G. (2020). Use of myo-inositol plus Bifidobacterium lactis and Lactobacillus rhamnosus for preventing gestational diabetes mellitus in Mexican women. Gac. Med. Mex. 156. 10.24875/GMM.M20000438 33373358

[B39] Shin M. Liu Q. F. Choi B. Shin C. Lee B. Yuan C. (2020). Neuroprotective effects of limonene (+) against aβ42-induced neurotoxicity in a *Drosophila* model of alzheimer’s disease. Biol. Pharm. Bull. 43, 409–417. 10.1248/bpb.b19-00495 31875578

[B40] Si L. H. Lin R. X. Jia Y. Jian W. W. Yu Q. Wang M. (2019). Lactobacillus bulgaricus improves antioxidant capacity of black garlic in the prevention of gestational diabetes mellitus: a randomized control trial. Biosci. Rep. 39. 10.1042/BSR20182254 PMC6689107 31362999

[B41] Siemelink M. Verhoef A. Dormans J. A. Span P. N. Piersma A. H. (2002). Dietary fatty acid composition during pregnancy and lactation in the rat programs growth and glucose metabolism in the offspring. Diabetologia 45, 1397–1403. 10.1007/s00125-002-0918-2 12378380

[B42] Song H. Chu Q. Yan F. Yang Y. Han W. Zheng X. (2016). Red pitaya betacyanins protects from diet-induced obesity, liver steatosis and insulin resistance in association with modulation of gut microbiota in mice. J. Gastroenterol. Hepatol. 31, 1462–1469. 10.1111/jgh.13278 26699443

[B43] Sweeting A. Wong J. Murphy H. R. Ross G. P. (2022). A clinical update on gestational diabetes mellitus. Endocr. Rev. 43, 763–793. 10.1210/endrev/bnac003 35041752 PMC9512153

[B44] Tian Z. H. Miao F. T. Zhang X. Wang Q. H. Lei N. Guo L. C. (2015). Therapeutic effect of okra extract on gestational diabetes mellitus rats induced by streptozotocin. Asian Pac J. Trop. Med. 8, 1038–1042. 10.1016/j.apjtm.2015.11.002 26706676

[B45] Tola A Faraj C. L. M. Erridge C. (2017). Host defenses against metabolic endotoxaemia and their impact on lipopolysaccharide detection. Int. Rev. Immunol. 3, 125–144. 10.1080/08830185.2017.1280483 28783409

[B46] Wang X. Liu H. Li Y. Huang S. Zhang L. Cao C. (2020). Altered gut bacterial and metabolic signatures and their interaction in gestational diabetes mellitus. Gut Microbes 12, 1–13. 10.1080/19490976.2020.1840765 PMC7714515 33222612

[B47] Wu N. Zhou J. Mo H. Mu Q. Su H. Li M. (2021). The gut microbial signature of gestational diabetes mellitus and the association with diet intervention. Front. Cell Infect. Microbiol. 11, 800865. 10.3389/fcimb.2021.800865 35096649 PMC8795975

[B48] Wu S. Wen F. Zhong X. Du W. Chen M. Wang J. (2023). Astragaloside IV ameliorate acute alcohol-induced liver injury in mice via modulating gut microbiota and regulating NLRP3/caspase-1 signaling pathway. Ann. Med. 55, 2216942. 10.1080/07853890.2023.2216942 37243569 PMC10228327

[B49] Wurtz P. Makinen V. P. Soininen P. Kangas A. J. Tukiainen T. Kettunen J. (2012). Metabolic signatures of insulin resistance in 7,098 young adults. Diabetes 61, 1372–1380. 10.2337/db11-1355 22511205 PMC3357275

[B50] Yao M. Zhang L. Wang L. (2023). Astragaloside IV: a promising natural neuroprotective agent for neurological disorders. Biomed. Pharmacother. 159, 114229. 10.1016/j.biopha.2023.114229 36652731

[B51] Ye G. Y. Zhang L. Wang M. Chen Y. B. Gu S. L. Wang K. Y. (2019). The gut microbiota in women suffering from gestational diabetes mellitus with the failure of glycemic control by lifestyle modification. J. Diabetes Res. 2019, 6081248. 10.1155/2019/6081248 31772944 PMC6854930

[B52] Zhang H. W. Qi C. Zhao Y. N. Lu M. Y. Li X. Y. Zhou J. B. (2021). Depletion of gut secretory immunoglobulin A coated Lactobacillus reuteri is associated with gestational diabetes mellitus-related intestinal mucosal barrier damage. Food Funct. 12, 10783–10794. 10.1039/d1fo02517a 34609395

[B53] Zhong Y. Liu W. Xiong Y. Li Y. Wan Q. Zhou W. (2022). Astragaloside Ⅳ alleviates ulcerative colitis by regulating the balance of Th17/Treg cells. Phytomedicine 104, 154287. 10.1016/j.phymed.2022.154287 35752072

