# Supplementary figures and images for: Astragaloside IV alleviates GDM via regulating gut microbiota and gut microbiota metabolomic
Source: Front Pharmacol. 2025 Jan 6;15:1431240. doi: 10.3389/fphar.2024.1431240 (PMC11780255; doi:10.3389/fphar.2024.1431240)

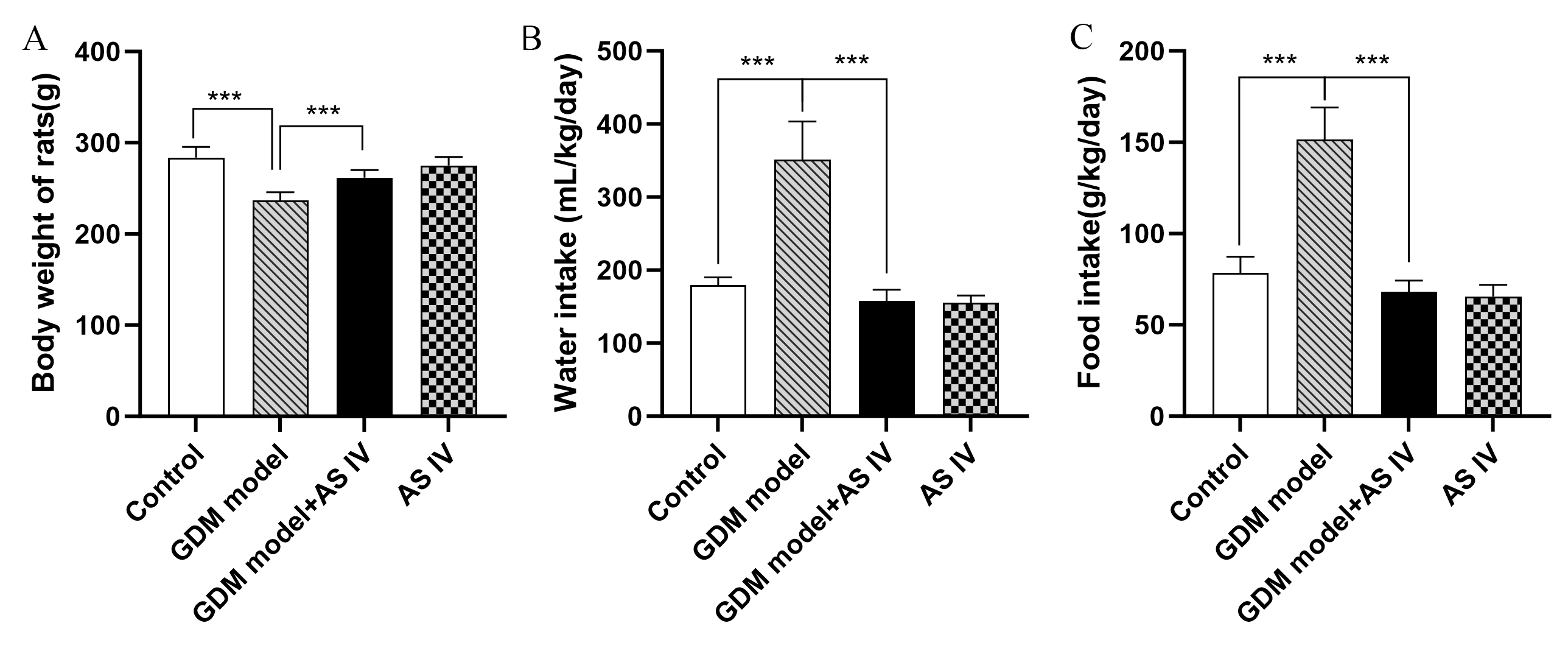

Supplement: Supplementary file 2 [file Image2.tif]

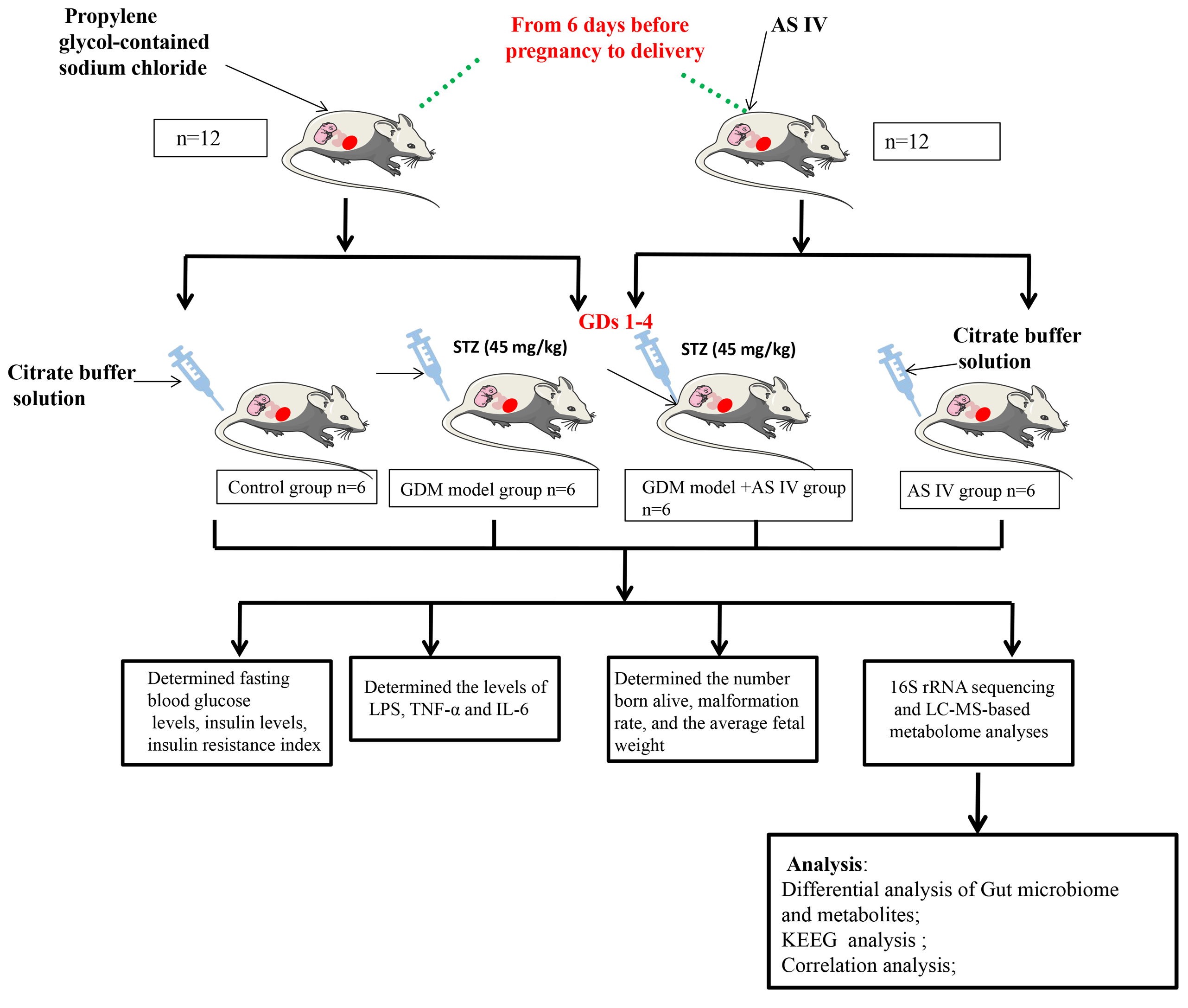

Supplement: Supplementary file 3 [file Image1.tif]
